# Molecular and Environmental Triggering Factors of Pathogenicity of *Fusarium oxysporum* and *F. solani* Isolates Involved in the Coffee Corky-Root Disease

**DOI:** 10.3390/jof7040253

**Published:** 2021-03-27

**Authors:** Roberto Gamboa-Becerra, Daniel López-Lima, Luc Villain, Jean-Christophe Breitler, Gloria Carrión, Damaris Desgarennes

**Affiliations:** 1 Red de Biodiversidad y Sistemática, Instituto de Ecología A.C. Carretera Antigua a Coatepec 351, El Haya, Xalapa, Veracruz 91073, Mexico; roberto.gamboa@inecol.mx (R.G.-B.); danielopez@uv.mx (D.L.-L.); 2 CIRAD, UMR DIADE, F-34394 Montpellier, France; luc.villain@cirad.fr (L.V.); Jean-christophe.breitler@cirad.fr (J.-C.B.)

**Keywords:** *Fusarium*, coffee corky-root disease, secreted in xylem genes, fumonisin genes, pathogenicity, *Fusarium solani*, water stress

## Abstract

Coffee corky-root disease causes serious damages to coffee crop and is linked to combined infection of *Fusarium* spp. and root-knot nematodes *Meloidogyne* spp. In this study, 70 *Fusarium* isolates were collected from both roots of healthy coffee plants and with corky-root disease symptoms. A phylogenetic analysis, and the detection of pathogenicity *SIX* genes and toxigenicity *Fum* genes was performed for 59 *F. oxysporum* and 11 *F. solani* isolates. Based on the molecular characterization, seven *F. oxysporum* and three *F. solani* isolates were assessed for their pathogenicity on coffee seedlings under optimal watering and water stress miming root-knot nematode effect on plants. Our results revealed that a drastic increment of plant colonization capacity and pathogenicity on coffee plants of some *Fusarium* isolates was caused by water stress. The pathogenicity on coffee of *F. solani* linked to coffee corky-root disease and the presence of *SIX* genes in this species were demonstrated for the first time. Our study provides evidence for understanding the pathogenic basis of *F. oxysporum* and *F. solani* isolates on coffee and revealed the presence of *SIX* and *Fum* genes as one of their pathogenicity-related mechanisms. We also highlight the relevance of chlorophyll, a fluorescence as an early and high-throughput phenotyping tool in *Fusarium* pathogenicity studies on coffee.

## 1. Introduction

The Meloidogyne-based disease complexes (MDCs) involve the interaction of different root-knot nematodes (RKN) *Meloidogyne* spp. and phytopathogenic fungi, especially *Fusarium* spp. which causes severe damage to several important crops worldwide including coffee [1,2,3,4]. In addition, specific bacterial communities are also involved in these MDCs pathosystems and are likely to be responsible for more severe symptoms [3,5]. 

In coffee plants, the MDCs cause severe symptoms known as corky-root disease, which lead to necrosis and atrophy of the root system. This is the case with *M. arabicida* in Costa Rica, *M. incognita* mainly in Brazil or *M. paranaensis* in Brazil, Guatemala, Hawai, and Mexico [1,2,4,6,7,8]. As described by these authors, the inefficient functioning of roots with reduced uptake of water and nutrients (like drought symptoms in the plant canopy) is due to diverting water and nutrients to the growing female nematodes in infected roots. Consequently, coffee plants present significant damage including chlorosis, defoliation, necrosis of tip branches, and reduced production. Bertrand et al. (2000) [1] demonstrated that only the combination of *Fusarium oxysporum* with *M. arabicida* produced corky-root symptoms on *Coffea arabica*. *Fusarium oxysporum* alone was non-pathogenic, and *M. arabicida* alone caused simple galls and reduction in shoot height, but no corky-root symptoms. Hua et al. (2019) [9] reported that the inoculation of *M. incognita* together with *F. oxysporum* f. sp. *niveum* enhanced the susceptibility of several watermelon genotypes to *Fusarium* wilt (including genotypes resistant to *F. oxysporum* f. sp. *niveum*) and led to an early development of wilt symptoms and increased disease damage.

Loópez-Lima et al. (2020) [4] found that all the 27 *F. oxysporum* isolated from the coffee corky-roots were able to colonize the vascular system of roots and some the stem of coffee seedlings whether the inoculation was carried out on wounded or intact roots. However, none of these isolates caused wilting symptoms on the coffee plants in the absence of the nematode *M. paranaensis*. The question remains whether these fungi are latent pathogens, opportunists, or saprophytes in coffee roots while coffee plants are not infected by nematodes or submitted to another type of stress especially abiotic stress. *Fusarium* species are well known as capable of changing their trophic lifestyles, transitioning between asymptomatic biotropic to destructive necotrophic phase (biotrophy-necrotrophy switch) depending of the environmental conditions, including several biotic and abiotic stresses [10,11].

*Fusarium* genus consist of a group of soilborne non-plant-pathogenic and plant-pathogenic strains, the later causing vascular wilt and root diseases on a broad range of economically important crops [12]. Nowadays, more than 120 *formae speciales* (f. sp.) have been described according to their pathogenicity to just one or few host plant species [13,14]. In many cases, the host-specificity of phytopathogenic fungi depends on a repertoire of effector genes encoding for virulence factors such as small secreted proteins and enzymes participating in the synthesis of host-specific toxins that interfere with the host plant immunity [15,16]. Effector proteins are secreted in initial plant defense response to play a range of functions that include promotion of host colonization, masking of the presence of the pathogen, suppression of host defense responses, and transcriptional reprogramming of the host cell [14,17]. In *Fusarium oxysporum species complex* (*FoSC*), only one family of effectors has been identified, the *Secreted In Xylem* (*SIX*) genes, which are located in lineage-specific mobile pathogenicity chromosomes [16]. Fourteen *SIX* genes have been identified in *F. oxysporum* f. sp. *lycopersici* (*Fol*) and were initially thought to be exclusive to *Fol*. However, several homologous genes have been detected in other *formae speciales* such as *betae*, *canariensis*, *cepae*, *ciceris*, *conglutinans*, *cubense*, *fragarie*, *lilii*, *medicaginis*, *melonis*, *niveum*, *passiflorae*, *pisi*, *radicis-cucumerinum*, *radices-lycopersici*, *raphani*, *vasinfectum*, and *zingiberi* [12,13,17,18,19,20,21,22,23].

The elucidation of the molecular basis involved in the pathogenicity of *Fusarium* species complex associated with coffee corky-root disease, remains unexplored. The aim of this study was to characterize the genetic variability of *Fusarium* associated with coffee corky-root disease, through analysis of housekeeping, toxigenicity, and pathogenicity-related genes, and parallel to test their pathogenicity alone or combined with water stress which can mimic one of the major direct effect of RKN infection i.e., less water availability for the plant. The *SIX*-*Fum* genes profiles of *Fusarium* isolates were compared with their capacity to cause disease symptoms in coffee plants.

## 2. Materials and Methods

### 2.1. Coffee Corky-Roots Sampling

Samples of roots of healthy coffee plants and with corky-root disease symptoms were obtained from 13 coffee plantations (localities) belonging to nine municipalities of the main coffee cropping area of the state of Veracruz, Mexico. The sampled sites were selected based on previous information [4] registering coffee plants with symptoms of corky-root disease. In every plantation, roots of 3–5 coffee plants were sampled and brought together to obtain one composite bulk of every sampling site. 

### 2.2. Obtention of Fusarium Isolates

The obtention of the *Fusarium* isolates from the 13 sampling sites was conducted by selecting roots of healthy coffee plants as well as roots with early symptoms of corky-root disease but without necrosis. Healthy and symptomatic plants were differentiated by visual inspection based in the absence (healthy coffee plants) or presence of the symptoms associated with coffee corky-root disease, which include main and secondary roots swelling with cracking aspect, visible nutritional deficiency symptoms of the plant, defoliation, chlorosis, necrosis of tip branches, and reduced production. In total, 70 *Fusarium* isolates were obtained, four isolates (CBF-244, CBF-254, CBF-263, and CBF-269) were obtained from roots of healthy coffee plants and 66 *Fusarium* isolates from roots of coffee plants with early symptoms of corky-root disease. Sampled roots were washed under tap water to remove excess soil and then surface disinfected by soaking them first in 70% ethanol (for 1 min), then in 3% NaCIO for 1 min, and in 96% ethanol for 30 s. Finally, root samples were washed three times with sterile distilled water. Longitudinal cuts were made on the root tissues and small pieces of at most 0.5 cm^2^ of the inner tissues were extracted and deposited on potato dextrose agar (PDA) Petri dishes containing 4 g/L potato infusion, 20 g/L dextrose, and 15 g/L agar, and chloramphenicol (1 mg mL^−1^). The plates were incubated at 25 °C during 4–7 days for the development of typical *Fusarium* mycelial growth and sub-cultured on PDA Petri dishes until obtaining pure cultures.

### 2.3. Molecular Characterization of Fusarium Isolates

#### 2.3.1. DNA Extraction

*Fusarium* isolates were grown for four days at 25 °C in potato dextrose broth (PDB) containing 4 g/L potato infusion and 20 g/L dextrose. For each isolate, 50–100 mg of mycelium were placed in 1.5 mL tubes containing sterile glass microspheres (<0.5 mm Ø), then 500 µL of extraction buffer, and 2.5 µL RNAse A (10 mg mL^−1^) were added [24]. Tubes were vigorous vortexed and incubated for 40 min at 45 °C. Afterwards, 150 µL of potassium acetate (5 M) were added and incubated on ice for 15 min. Then, tubes were centrifuged at 12,000 rpm for 5 min and the supernatant transferred to a clean tube. DNA precipitation was made adding 500 µL of cold isopropanol and incubating at least for 2 h at −20 °C, and centrifuging at 12,000 rpm during 5 min. Supernatants were discarded, the DNA pellet was washed with 500 µL of cold ethanol, and centrifuged for 5 min. Finally, the pellet was resuspended in 50 µL of sterile miliQ water. 

#### 2.3.2. Phylogenetic Analysis

The translation elongation factor (*EF-1α*), RNA polymerase II second largest subunit (*RPB2*), and β-tubulin (*TUB2*) genes were selected to infer phylogenetic relationships between *Fusarium* isolates. Amplifications by PCR of the three housekeeping genes were conducted employing previously published primers [12,25,26]. Thermocycling conditions were as follows: one cycle of 3 min at 95 °C; 40 cycles of 1 min at 95 °C, 30 s for alignment (67, 59, and 61 °C for *EF-1α*, *RPB2*, and *TUB2*, respectively) and 1 min at 72 °C; finally, extension time of 10 min at 72 °C. PCR amplifications were performed in a Bio-Rad thermal cycler (T100^TM^) in 25 µL reactions containing 1 U of Taq DNA Pol (Qiagen), buffer 10× (MgCl_2_ 15 mM), a final concentration of 0.5 µM of each primer, DNTPs mix (10 mM), 1 µL (~100 ng) of template DNA, and 19.3 µL of nuclease-free water. PCR products of 1269, 881, and 1500 bp were obtained for *EF-1α*, *RPB2*, and *TUB2*, respectively. The identification of the *Fusarium* isolates was performed in NCBI BLAST algorithm (https://blast.ncbi.nlm.nih.gov/Blast.cgi/ (accessed on 30 November 2020)) by comparison of *EF-1α* sequences against NCBI database. Amplicons were purified using the Wizard SV PCR and Gel Clean-Up kit (Promega) and sent for sequencing (Macrogen INC) using forward and reverse primers. The obtained sequences were edited, aligned by ClustalW, and concatenated using MEGA version 7.0.26 [27], the phylogenetic tree was generated through maximum likelihood method based on the best model Hasegawa–Kishino–Yano (HKY + G) using concatenated *EF-1α*, *RPB2*, and *TUB2* data supported by 1000 bootstrap replicates. DNA Sequences of *Fusarium* genus deposited on Gene Bank were included as references. The tree was rooted through the outgroup *Fusarium dimerum*.

#### 2.3.3. PCR Analysis of SIX Genes

Molecular pathogenicity characterization was made through the detection (presence/absence) of putative effector *SIX*1–*SIX*14 genes using primers previously reported [12,17,28,29]. All PCR amplifications for *SIX*1–*SIX*14 genes were conducted as mentioned for housekeeping genes. Thermocycling conditions were as follows: first, 3 min at 95 °C; 35 cycles of 1 min at 95 °C, 30 s for annealing (at 57 °C for *SIX*1, 60 °C for *SIX*2 and *SIX* 8, 59 °C for *SIX3*–*SIX* 7, 67 °C for *SIX* 9, and 64 °C for *SIX1*0–*SIX1*4) and 1 min at 72 °C. Final extension of 10 min at 72 °C. Amplicons were visualized on 1.25% agarose gel stained with gel red.

#### 2.3.4. Molecular Detection of Putative Toxigenic *Fusarium* Isolates

In order to identify potentially toxigenic *Fusarium* isolates, primers previously published [28,29] were employed by targeting *Fum1* and *Fum 13* genes for detection of fumonisin producing isolates. Reaction composition for amplifications were carried out as mentioned before, the PCR conditions were as follows: an initial denaturation at 95 °C for 3 min, 30 cycles of 95 °C for 1 min, 30 s min at 59 and 61 °C (for *Fum* 1 and *Fum*13, respectively), and 72 °C for 1 min, with a final extension of 10 min at 72 °C. PCR products were loaded and visualized onto 1.25% agarose gel containing GelRed Nucleic Acid Gel Stain (Biotium, Fremont, CA, USA).

### 2.4. Pathogenicity Testing in Coffee Seedlings 

For pathogenicity test, seeds of *C. arabica* cv. Bourbon were germinated on sterile vermiculite at 28 °C in a growth chamber. After germination, the seedlings were transferred to individual tube-like pots with sterile vermiculite. Groups of 10 tube-like pots were placed in separate supporting containers to avoid contamination between treatments. Seedlings were grown at 25 °C with a 12 h photoperiod in a growth chamber. The selected *Fusarium* isolates were growth in PDB to obtain a solution of 1 × 10^6^ spores/mL. Then, 1 mL of that solution was inoculated in the stem base of each coffee seedling with three pairs of leaves (*n* = 10 plants/per treatment). The experiment was performed in two conditions: normal and under water stress. For the water-stress condition, the watering regime was changed, instead to water the seedlings to field capacity every other day (normal condition), they were watered every 6 days to get water stress. Peters ™ 9-45-15 water-soluble fertilizer was applied at a dose of 0.2 g per plant/week in the volume of water corresponding to each irrigation condition. Forty-five days after inoculation, coffee seedlings were removed from their containers and plant disease damage was assessed based on a symptom damage scale of 1–5 according to Reis and Boiteux (2007) [30]: 1 = plant free of symptoms, 2 = plant without wilting symptoms, but with light brown spots on the root, 3 = plants with vascular necrosis symptoms, wilting symptoms and slight yellowing, 4 = severe wilting associated with the presence of foliar necrosis and chlorosis, and 5 = dead plant. Aerial, root, and total fresh weights, plant height, and leaves number were measured.

### 2.5. Chlorophyll a Fluorescence Measurement in Coffee Seedlings 

Chlorophyll *a* fluorescence was measured as an indicator of the physiological status as well as the photosynthetic stress level of the coffee seedlings in response to both biotic and abiotic stresses. Chl *a* fluorescence transient of 25 min dark-adapted attached coffee mature leaves (L3) were measured between 08:00 to 11:00 am with a Handy-PEA^®^ chlorophyll fluorimeter (Handy-Plant Efficiency Analyser, Hansatech Instruments, King’s Lynn, Norfolk, UK). Measurements were performed 30 times on different seedlings per condition. Every measurement was performed on apparently healthy leaves, fully light-exposed. Chlorophyll *a* fluorescence transient was induced by 1 s illumination with an array of six light-emitting diodes providing a maximum light intensity of 3000 PAR. Fluorescent transients were analyzed using the JIP test developed by Strasser and Strasser (1995) [31], which evaluates the balance between total energy inflows and outflows and provides the probable distribution of light energy absorption (ABS) between the events: trapping (TR), electron transport (ET), and dissipation (DI). Some parameters were analyzed in more detail: the maximum quantum yield of photosystem II (FV/FM = TR/ABS), which expresses the trapping flux/absorption flux. This describes the performance of the light reaction; the energy flux transmitted per RC of PSII (TR/RC); the energy flux dissipated per RC (DI0/RC); the energy flux transported per RC (ET/RC) which represent the flux of electrons transported from QA to QB; and the “performance indices” (PIs) which combine information on the performance of PSII and efficiencies of specific electron transport reactions in the thylakoid membrane during the O-J-I-P rise [32]. These parameters provided information about specific and phenomenological fluxes, quantum yields, or “vitality” indexes, and permitted us to quantify the photosystems behavior in seedlings submitted to different treatments.

### 2.6. Re-Isolation and Identification of Fusarium Isolates by PCR Amplification 

After 45 days of inoculation of *Fusarium*, isolates in coffee seedlings, tissue fragments of basal and apical zones of root and stem tissues were recovered. The tissue fragments were cut into small pieces and were disinfected as mentioned above, then they were placed on PDA plates to obtain mycelial growth. Finally, *Fusarium* isolates were re-isolated to confirm their presence by amplification of either *EF-1α* or *RPB2* molecular markers. The obtained sequences were compared with the sequences previously determined in this study.

### 2.7. Statistical Data Analysis 

Data analysis was performed in RStudio (Version 1.3.1073) by using the vegan, ComplexHeatmap, corrplot, scatterplot3d, FactoMineR, and factoextra R packages. An analysis of variance (one-way ANOVA) with Tukey’s post hoc test was used to compare control and treatments for the measured variables. Statistically significance differences were detected at *p* < 0.05. The three-dimensional non-metric multidimensional scaling (3D-NMDS) was generated based on the Jaccard dissimilarity matrix of binary data (presence/absence of *SIX* and *Fum* genes). A permutational multivariate analysis of variance (PERMANOVA) with 999 permutations was conducted to evaluate the influence of species, municipality, and locality on the *SIX*-*Fum* genes profile of the *Fusarium* isolates. Principal component analysis (PCA) was conducted to test the discriminant power of quantitative photosynthetic variables to differentiate between pathogenic and non-pathogenic *Fusarium* isolates. A correlation analysis was conducted between the disease damage and the measured variables. Spearman’s rank correlation was used for the mixed data: an ordinal variable (disease damage) and all the continuous variables measured. Spearman correlation coefficient s(SCC) are reported at *p* < 0.01.

## 3. Results

### 3.1. Identification of Fusarium Isolates

Seventy isolates were identified based on their morphology and their *EF-1α* DNA sequences (Table 1). The highest percentage of *Fusarium* isolates (84.3%) corresponded to *Fusarium oxysporum*, 17 of the isolate sequences presented high identity against *formae speciales* of *F. oxysporum.* Of these, 11 corresponded to *F. oxysporum* f. sp. *vasinfectum*, three matched with *F. oxysporum* f. sp. *dianthi*, two corresponded to *F. oxysporum* f. sp. *phaseoli*, and one belonged to *F. oxysporum* f. sp. *cepae*. Interestingly, 11 isolates (15.7%) of this *Fusarium* complex isolated from coffee seedlings with the corky root disease were identified as *F*. *solani*.

### 3.2. Phylogenetic Analysis of Fusarium Isolates

**Figure 1:**
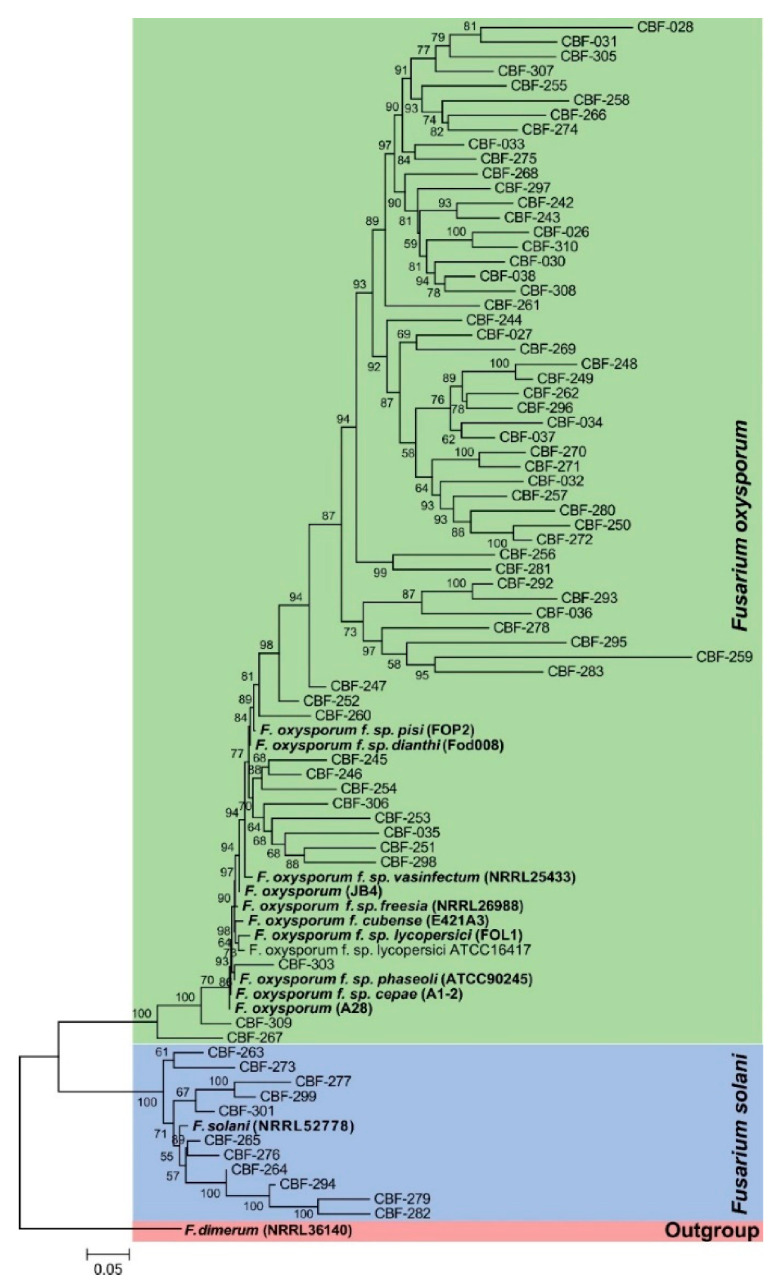
Phylogenetic tree of *Fusarium* isolates associated to coffee corky-root disease. The maximum likelihood tree was inferred from the concatenated *EF-1α*, *RPB2*, and *TUB2* genes sequence data set of the 70 isolates, based on the best model Hasegawa–Kishino–Yano (HKY + G), bootstrap values (*n* = 1000). The bar indicates 0.05 substitution per site. The tree is rooted through the outgroup *Fusarium dimerum* (NRRL36140). Reference sequences from *Fusarium*-ID database and GenBank included in the analysis are highlighted in bold letters.

To determine the genetic diversity of the *Fusarium* isolates associated with coffee corky-root disease and their relationships, we generated separated phylogenetic trees based on the single sequences of *EF-1α*, *RPB2*, and *TUB2*, which recovered similar topologies and clade support (data not shown) compared to the combined sequence dataset, which is presented in Figure 1. In this figure, concatenated phylogenetic tree comprising *EF-1α*, *RPB2*, and *TUB2* sequences showed the formation of two main clades separating *F. solani* from *F. oxysporum*. Isolates CBF-303 and CBF-267, which showed high *EF-1α* identity against *F. oxysporum* f. sp. *phaseoli*, and CBF-309 were clustered together with reference sequences of *F. oxysporum* f. sp. *phaseoli* and *F. oxysporum* f. sp. *cepae*. The majority of the *F. oxysporum* isolates were clustered in a large clade together with the closely related *F. oxysporum* f. sp. *pisi*, *F. oxysporum* f. sp. *dianthi,* and *F. oxysporum* f. sp. *vasinfectum*. The 11 isolates identified as *F. solani* were aligned in a separated clade with the reference sequence of *F. solani* NRRL52778 (JF740846.1)

### 3.3. Pathogenicity and Toxigenic Genes Characterization: SIX1–SIX14 and Fum Genes

In the *Fusarium* isolates CBF-244 (healthy plants isolate), CBF-253, CBF-301, and CBF-310 no *SIX* genes were detected. *SIX10*, *SIX12*, and *SIX13* genes were absent in all *Fusarium* isolates. *SIX11* gene was present only in CBF-263 isolate as it was also in the ATCC 417 strain. *SIX1* and *SIX8* were the most represented pathogenicity genes with a presence in 44.3% of total isolates, followed by *SIX7* with 37.1%. Interestingly, the CBF-263 isolate from healthy coffee plants contained six *SIX* genes: *SIX3*, *SIX4*, *SIX8*, *SIX9*, *SIX11*, and *SIX14* (Table 1).

Regarding toxigenicity genes, *Fum1* and *Fum13* were present always together in 27 of the studied *Fusarium* isolates (38.6%). One isolate, CBF-301, presented *Fum1* and *Fum13* genes but no *SIX* genes. Additionally, *Fum1* and *Fum13* were present in CBF-263 and CBF-269 isolates from healthy coffee plants (Table 1).

**Figure 2:**
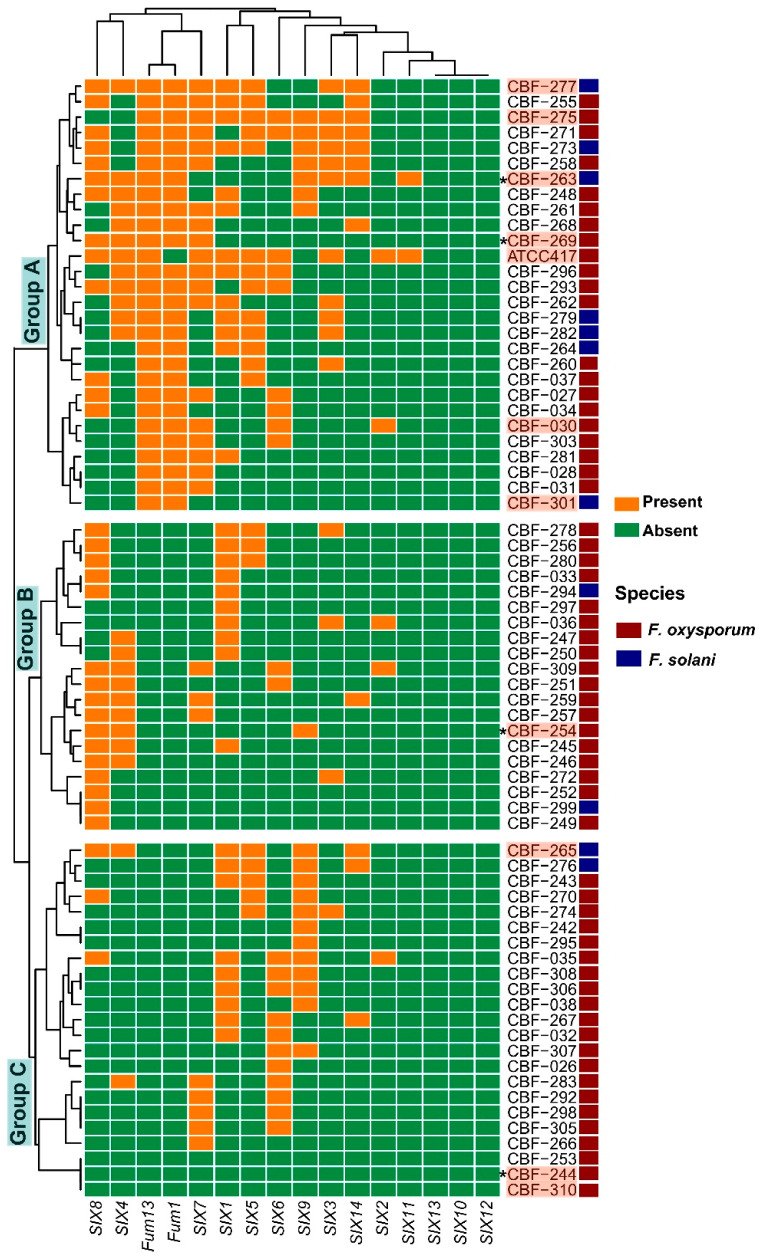
Binary heatmap of *SIX* and *Fum* genes (presence/absence) identified in the *Fusarium* isolates. The binary distance matrix was used to perform a hierarchical clustering with ward.D2 method. Presence, orange; absence, green. The *Fusarium* isolates used for pathogenicity test are highlighted. *Fusarium* specie are indicated by red (*F. oxysporum*) and blue squares (*F. solani*). The asterisks (*) indicate *Fusarium* isolates from healthy coffee seedlings.

Clustering analysis of isolates based in *SIX*-*Fum* genes profiles (presence/absence) evidenced the formation of three big groups (Figure 2). All isolates belonging to group A, and only these ones, contained *Fum*1 and *Fum*13 genes. In group A, those isolates with the highest number of *SIX* genes were also found: CBF-263, CBF-271, CBF-273, CBF-275, and CBF-277 isolates. Additionally, seven of the eleven *F. solani* isolates (CBF-263, CBF-264, CBF- 273, CBF-277, CBF-279, and CBF-282) were clustered in group A. Group A included the ATCC417 strain. In addition to the absence of toxigenicity *Fum* genes, group B was defined by the absence of *SIX11* gene and low frequencies of presence of *SIX6*, *SIX7*, *SIX9*, and *SIX14* compared to isolates clustered in group A. The group C shared with the group B the absence of *Fum* and *SIX11* genes, it was characterized by low frequencies of presence of *SIX8* and *SIX14*. No correlation was found between the *SIX* genes repertoire and the relationships of the *Fusarium* isolates observed across the phylogenetic tree (Figure 1 and Figure 2).

We performed a three-dimensional non-metric multidimensional scaling (3D-NMDS) analysis based on the binary data (presence/absence of the *SIX* genes) by using Jaccard dissimilarity index to visualize the grouping of the *Fusarium* isolates. The stress value obtained for the overall dataset was 0.10, the 3D-NMDS plots were divided in three figures to avoid the overlapping of the multiple variables related to municipality and locality (Figure S1a–c). PERMANOVA analysis revealed that the *SIX* gene profiles of the *Fusarium* isolates were influenced by the species, i.e., *F. oxysporum* vs. *F. solani* (*F* = 3.58, R^2^ = 0.05, *p* < 0.002) as illustrated in the 3D-NMDS plot (Figure S1a). The *SIX*-*Fum* genes profiles of *Fusarium* isolates were also affected by the geographical origin factors, municipality (*F* = 2.29, R^2^ = 0.23, *p* < 0.001) and locality (*F* = 1.84, R^2^ = 0.27, *p* < 0.001), although the clustering of samples was not so clear (Figure S1b–c). Our results suggest that the species and geographical origin influence the *SIX*-*Fum* genes profiles of *Fusarium* isolates associated to coffee corky-root disease.

### 3.4. Relationship between Global Disease Damages and the SIX and Fum Gene Repertoires of Fusarium Isolates

Based on screening and clustering analysis of pathogenicity and toxigenicity genes, we selected 10 isolates of *F. solani* and *F. oxysporum* to test their pathogenicity in planta: (a) CBF-244 (isolated from healthy plants) and CBF-310, in which no *SIX* and no *Fum* genes were detected; (b) CBF-301 isolate, which contained *Fum1* and *Fum13* genes, but did not contain *SIX* genes; (c) CBF-254 and CBF-265 isolates which did not contain *Fum* genes but contained three *SIX* (*SIX4*, *SIX8*, and *SIX9*) and six *SIX* genes (*SIX*1, *SIX*4, *SIX*5, *SIX*8, *SIX*9, and *SIX*14), respectively; (d) CBF-269 (isolated from heathy coffee plants) containing three *SIX* genes (*SIX4*, *SIX7*, and *SIX8*) and the two *Fum* genes; (e) CBF-263, CBF-275, and CBF-277 which contained the two *Fum* genes and they are among the isolates with the largest number of *SIX* genes; (f) CBF-030 which was the only one in which was detected the combination of *SIX2*, *SIX6*, and *SIX7* genes in addition to containing *Fum1* and *Fum13* toxigenicity genes; and (g) ATCC 417 (*Fusarium oxysporum* f. sp. *lycopersici*) was included to determine if despite its host specificity, it was able to elicit disease symptoms in coffee seedlings, considering its well-known pathogenicity level in tomato and its repertoire of *SIX* genes. In this study, the presence of *SIX1*–*SIX7* was confirmed for ATCC 417 and additionally, we detected amplification for *SIX8* (being the only isolate containing this *SIX* gene), *SIX11*, and *Fum13* genes (Table 1, Figure 2).

**Figure 3:**
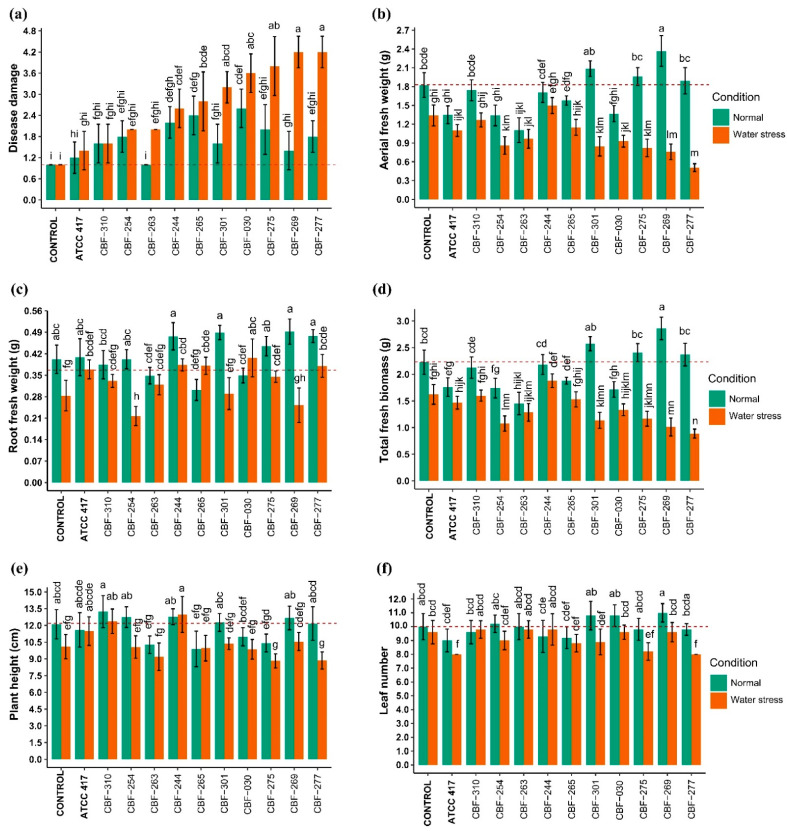
Effects of *Fusarium* isolates on disease damage and agronomical parameters of coffee seedlings. (**a**) Disease damage; (**b**) aerial fresh weight; (**c**) root fresh weight; (**d**) total fresh weight; (**e**) plant height; (**f**) leaf number. Data points represent mean ± SD (*n* = 5). Treatments sharing one or more letters are not significantly different (*p* < 0.05). Red dotted lines indicate control average.

Our results indicated that the isolates CBF-310, CBF-254, CBF-263, and the strain ATCC 417 (*Fusarium oxysporum* f. sp. *lycopersici*) did not cause significant level of disease compared to the control, with or without water stress (Figure 3a). The analysis of variance with Tukey’s post hoc test showed that without water stress, three isolates, CBF-244, CBF-265, and CBF-030 caused higher disease damages compared to the control (*p* < 0.01). These three isolates also caused higher disease damages under water stress compared to the control although not significantly higher than under no-water-stress conditions (*p* < 0.01). The results obtained for the isolate CBF-244 were surprising because it was characterized by the absence of both *SIX* and *Fum* genes, whereas in the isolate CBF-265, *Fum1* and *Fum13* genes were absent, but it contained *SIX*1, *SIX*4, *SIX*5, *SIX*8, *SIX*9, and *SIX*14. Finally, though they did not cause significantly higher disease damages compared to the control in absence of water stress, four isolates caused significantly higher damages of disease under water stress: CBF-301 and especially CBF-275, CBF-269, and CBF-277, which showed the highest disease damage scores (4.2) (Figure 3a). Interestingly, all these *Fusarium* isolates were clustered in Group A of the *SIX* and *Fum* gene presence/absence heatmap (Figure 2) containing all and only the isolates carrying the *Fum1* and *Fum13* genes. Four of the isolates producing the most severe symptoms of disease in coffee seedlings, CBF-030, CBF-269, CBF-275, and CBF-277, were the only ones containing the *SIX7* gene.

### 3.5. Effects of Fusarium Isolates Inoculation on Coffee Seedling Growth 

Aerial (AFW), root (RFW), and total fresh weights (TFW), plant height (PH), and leaf number (LN) of coffee seedlings were measured in response to inoculation of selected *Fusarium* isolates under and without water-stress conditions.

We found that the isolates CF-244 and CBF-310 did not cause significant changes in any of the measured plant growth variables, with or without water stress (Figure 3b–f). Our results showed that the strain ATCC 417 and the isolates CBF-254, CBF-263, and CBF-030 decreased the AFW under no-water-stress conditions. The last three isolates also diminished AFW under water stress, but for CBF-254 and CBF-030, the AFW was significantly lower compared to the no-water-stress conditions (*p* < 0.01). The isolates CBF-301, CBF-275, CBF-269, and CBF-277 were not able to decrease the AFW under non-water-stress conditions, but under stress conditions they caused a reduction of up to 72% in AFW values as was the case of the isolate CBF-277 (Figure 3b). The RFW was only reduced by the isolate CBF-254 under stress conditions (Figure 3c). The isolates CBF-263 and CBF-030 were the only ones that declined the TFB values under no-water-stress conditions compared to the control, but no effect was observed for these isolates under water stress condition. On the contrary, although the isolates CBF-254, CBF-301, CBF-275, CBF-269, and CBF-277 did not cause significantly lower TFB values in absence of water stress, these isolates negatively affected the TFB under water stress (Figure 3d). The isolate CBF-265 was the only one that decreased the height of coffee seedlings under no-water-stress conditions (Figure 3e). Finally, we found that leaf number of coffee seedlings was only reduced under stress conditions by the strain ATCC 417 and the isolates CBF-275 and CB-277 (Figure 3f).

### 3.6. Effects of Fusarium Isolates Inoculation on Photosynthetic Activity of Coffee Seedlings 

**Figure 4:**
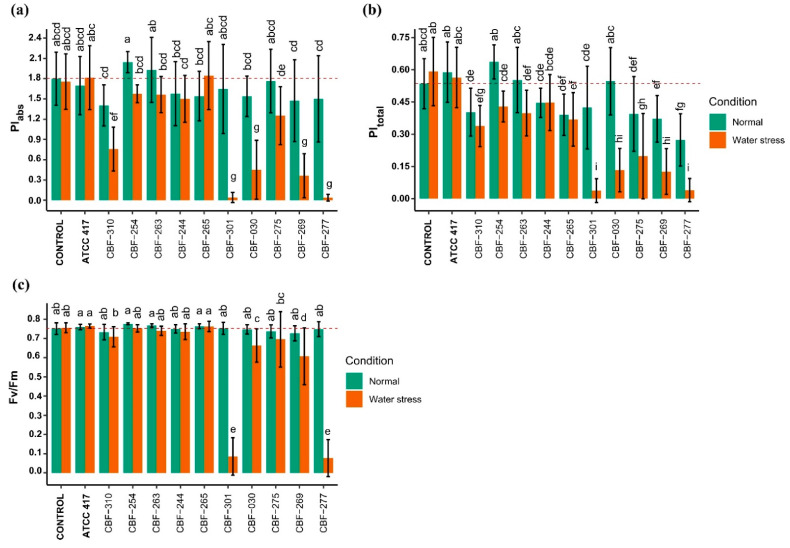
Effects of *Fusarium* isolates on photosynthetic activity indicators. (**a**) PI_abs_; (**b**) PI_total_; (**c**) Fv/Fm. Data points represent mean ± SD of at least twenty measurements of ten coffee plants. Treatments sharing one or more letters indicate are not significantly different (*p* < 0.05). Red dotted lines indicate control average.

The ANOVA analysis with Tukey’s post hoc test demonstrated that the isolates CBF-254, CBF-263, and CBF-244 did not influence the variables PI_abs_, PI_total_, and Fv/Fm either in no-water-stress or under stress conditions. Whereas on the contrary, a drastic decline (*p* < 0.01) of those three fluorescence parameters was caused by the isolates CBF-301, CBF-030, CBF-269, and CBF-277 under water stress conditions (Figure 4a–c). 

The energy fluxes in the energy cascade in PSII for the events absorption (ABS), trapping (TR_0_), electron eransport (ET_0_), and dissipation (DI_0_), under water stress, were also affected by these *Fusarium* isolates, displaying a high level of energy absorption (ABS/RC), a lower level of trapping (TR_0_/RC) and electron transport (ET_0_/RC) combined with a high level of energy dissipation (DI_0_/RC) (data not shown). The isolates CBF-269 and CBF-277 also decreased the PI_abs_ values in absence of water stress but those values were significantly lower under water stress conditions (Figure 4b). These results were in accordance with those observed for the global disease damages and the plant growth variables, since all those isolates (CBF-301, CBF-030, CBF-269, and CBF-277) were found to be among the most pathogenic isolates.

**Figure 5:**
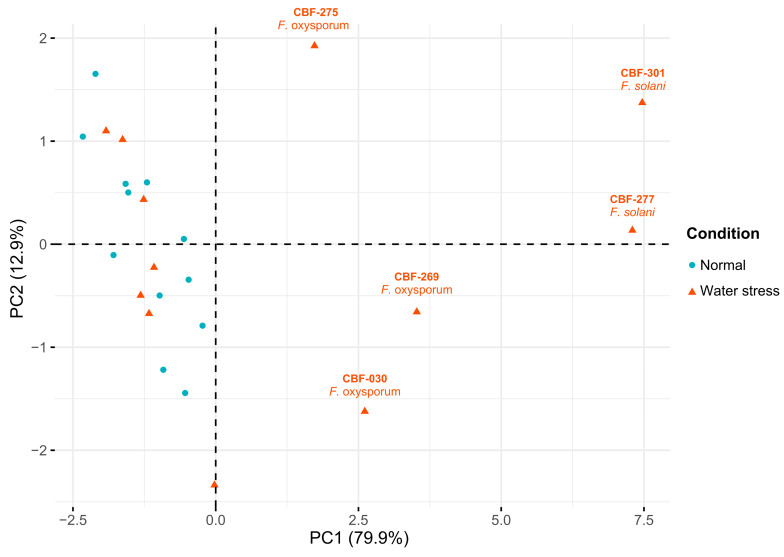
Principal component analysis (PCA) of *Fusarium* isolates based in photosynthetic parameters. PCA analysis was generated with the quantitative data of measurements PI_total_, PI_abs_, phi(Eo), Fv/Fm, ABS/RC, Dio/RC, TRo/RC, Eto/CSo, and ψ0/Vj. The first and second PC together account for 92.8% of discriminant power.

In accordance with the PI_abs_ and PI_total_ results, the extreme decrease of Fv/Fm observed on coffee seedling inoculated with CBF-301 and CBF-277 and under water stress attest to irreversible physiological damage on these plants. A principal component analysis (PCA) was performed by employing the data of measurements of all photosynthetic variables. The first two principal components PC1 and PC2 explained 92.8% of the total variance (Table 2). The PC1 accounted for 80% of the variation and was correlated (*r* > 0.9) with most of the photosynthetic parameters except for Tr_0_/RC (*r* = −0.31). The clustering of *Fusarium* isolates was dependent of the photosynthetic status, which showed a clear discrimination of the five most pathogenic isolates: CBF-030, CBF-275, CBF-269, CBF-277, and CBF-301 (Figure 5).

### 3.7. Correlation between Disease Damage and the Phenotypic Variables Assessed in Coffee Seedlings

To investigate the relationship between disease damage and each of the phenotypic variables, a correlation analysis was conducted using Spearman’s rank correlation, with a significance at *p* < 0.01. Associations with Spearman correlation coefficient (SCC) values of 0.4–0.59 were considered as “moderate” correlations, 0.60–0.79 as “strong” correlations and values greater than 0.8 corresponded to “very strong” correlations. Photosynthetic activity indicators PI_abs,_ PI_total_, and Fv/Fm were the variables which showed the strongest correlation with the damge of the disease (Figure S2). Contrary to the plant growth variables, leaf number, plant height, and aerial fresh weight, for which negative correlations were observed (SCC: −0.41, −0.51 and −0.6, respectively) in relation to the disease damage.

The photosynthetic parameters were strong to very strong correlated with each other (SCC: 0.73 to 0.89). PI_abs_, PI_total_, and Fv/Fm were positively correlated with AFW (SCC: 0.42, 0.52, and 0.49, respectively), whereas LN vs. Fv/Fm and PH vs. PI_total_ showed moderately positive correlations. Likewise, positive correlations were observed between all growth variables AFW, LN, PH, and RFW (Figure S2).

### 3.8. Vascular Colonization of Fusarium Isolates in Aerial and Root Tissues of Coffee Seedlings

**Figure 6:**
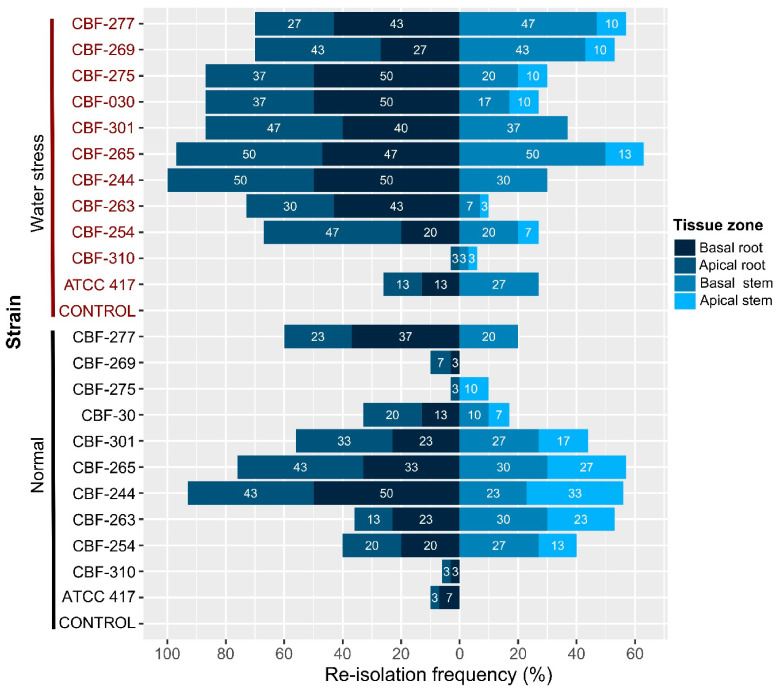
Re-isolation frequency of *Fusarium* isolates from roots and stem tissues after 45 days of post-inoculation. *Fusarium* isolates were reisolated of basal and apical zones of both root and stem tissues. The identification of re-isolates was determined by amplification and sequencing of either *EF-1α* or *RPB2* gene. Sequences were compared against sequences obtained previously for all the isolates.

Most of the isolates inoculated in pathogenicity tests were re-isolated in percentages lower than 40% in root tissues under normal conditions (Figure 6). In general, the colonization by the *Fusarium* isolates was increased under water-stress conditions in both basal and apical zones of roots.

Interestingly, the isolates CBF-265 and CBF-244, which induced symptoms of disease in coffee seedlings without water stress, colonized in higher percentages (76 and 93%, respectively) the root tissue in absence of water stress. The CBF-030 isolate, which also caused disease symptoms under absence of water stress, colonized the root tissue in 33% but the percentages increased up to 87% under water stress condition.

Low colonization capacity of isolate CBF-310 was in accordance with the fact that it did not produce symptoms of disease either in normal or under water-stress conditions like the ATCC-417 strain. The isolates CBF-275, CBF-269, and CBF-277 characterized by producing the strongest negative effects on photosynthetic activity and growth of coffee seedlings under water stress, were also able to colonize both roots and stem tissues in high percentages under water-stress conditions (Figure 6). In general, our results indicated that: the percentages of colonization in root were higher compared to stem tissue; for some isolates, the colonization percentages were higher under water stress compared to normal conditions; and there was a global relationship between the observed disease damage and the colonization capacity of *Fusarium* isolates.

## 4. Discussion

### 4.1. Diversity of Fusarium Linked to Coffee Corky-Root Disease

*Fusarium* spp. have been reported as linked to coffee corky-root disease in association with the root-knot nematodes *Meloydogine arabicida* and *M. paranaensis* [1,7,8,33]. Among the diversity found in this studied *Fusarium* complex, 59 isolates belonged to *F. oxysporum* while only 11 isolates belonged to *F. solani*. These results are in accordance with the observations of López-Lima et al. (2020) [4] who found that *F. oxysporum* was the dominant fungal species in coffee corky-root samples. Molecular markers flanking the internal transcriber spacer (ITS) have been used to determine the identity of *Fusarium* isolates linked to coffee corky-root disease [4]. In this study, phylogenetic analysis using concatenated sequences of three different markers *EF-1α*, *RPB2*, and *TUB2* allowed the identification of *Fusarium* isolates with high similarity against different *formae speciales* of *F. oxysporum* which corresponded to f. sp. *vasinfectum*, f. sp. *dianthi*, f. sp. *phaseoli*, and f. sp. *cepae*. Nevertheless, interestingly 45 of the 59 *Fusarium* isolates were grouped in a well-supported branch separated of the other *formae speciales* and *F. oxysporum* sequences included as references. Further research should focus on clarifying how the diversity of *Fusarium* isolates interact for the development of corky-root disease.

### 4.2. Isolates of F. oxysporum and F. solani Causing Disease Symptoms in Coffee Seedlings under Water Stress

In this study, we demonstrated that some *F. oxysporum* and *F. solani* isolates from coffee seedlings produced vascular wilting, chlorosis, tissue necrosis, and ultimately plant death, but without producing corky-root symptoms in absence of nematodes, and especially under water stress. Bertrand et al. (2000) [1] demonstrated that corky-root symptoms are exhibited only under the combined infection by the nematode *M. arabicida* and a *Fusarium oxysporum* isolate. The lack of pathogenicity of the studied *Fusarium* isolates in absence of nematodes or of an abiotic stress indicates that resistance to only *M. paranaensis* seems to be a good strategy for controlling the coffee corky-root disease. This has been confirmed by the observations in fields infected by *M. paranaensis* when planting susceptible Arabica cultivars grafted on *C. canephora* cv. Nemaya rootstock resistant to *M. paranaensis* [2].

It was found that two *F. solani* isolates were among the five isolates producing the strongest disease symptoms under water stress, which is a novel finding in the etiology of the coffee corky-root disease. There is only two reports from Kenya and Yemen in which *F. solani* was determined as a causal agent of a wilting disease but not of a corky-root disease in coffee [34,35].

Our results indicated that under water stress, some *Fusarium* isolates became very pathogenic on coffee seedlings, resulting in severe symptoms with wilting and a severely affected photosynthetic activity which results in a strongly affected plant growth. It was also observed that plant colonization by these *Fusarium* isolates increased under water stress. The underlying mechanisms leading to such severe physiological disruption of coffee plants under the combined action of some *Fusarium* and water stress have yet to be explained. However, we can hypothesize that pathogenic *Fusarium* highly reduce water flow in the plant by blocking the xylem vascular system, which has been demonstrated for many crops [36,37,38,39,40]. This work highlights that climate change that is increasing the frequency and intensity of drought periods in coffee crop [41], could lead to a greater impact of some *Fusarium* on coffee even in the absence of RKN as observed in Kenya and Yemen [34,35].

The synergy between abiotic and biotic stress-crop interactions has showed to induce changes in host physiology leading to changes on vegetative variables, deficit in nutritional status, increased susceptibility, as well as dysregulation in expression of photosynthetic genes [42,43,44,45,46]. Under abiotic and biotic stress, ROS are produced affecting photosynthetic electron transport, impairing the assembly and repair of PSII and affecting chloroplast development [47,48]. Depression of photosystems II and I performance has been observed either in the case of just wilting fungi infections [49,50,51].

In this study, the decreased efficiency of energy trapping in PSII reaction centers (Fv/Fm) observed for those isolates, decreased values of performance indices (PIs) combined with high level of photon flux absorption, low level of electron transport, and high level of energy dissipation indicated very low photosynthetic yield, probably irreversible photosystem damage and significant oxidative stress.

Chlorophyll *a* fluorescence and especially performance indices are used to estimate photosynthetic stress level, particularly in the case of abiotic stress and more rarely to evaluate biotic stress or a combination of both [52]. Toniutti et al. (2017) [53] demonstrated that parameters related to Photosystem II and photosynthetic electron transport chain components are powerful indicators of the physiological status of the coffee plants and predict infection intensity of *Hemileia vastatrix* in combination with abiotic stress.

In our case study, the use of quantitative data of the photosynthetic parameters was able to well discriminate between the non-pathogenic and the pathogenic *Fusarium* isolates, and especially the parameters Fv/Fm and PI_abs_,_total_ proved to be the best indicators of the disease damage. Here, we demonstrated that chlorophyll *a* fluorescence could be used as a fast and precise assessment of the level of damage comparatively to growth evaluation.

### 4.3. The Repertoire of SIX-Fum Genes Is Associated with Pathogenicity of Fusarium Isolates

At present, there are no reports about the characterization of presence of the *SIX* genes and the *Fum* genes in *Fusarium* isolates of coffee plants. A significant characteristic of all *Fusarium* isolates from coffee seedlings, either healthy or with corky-root symptoms, was the absence of *SIX10*, *SIX12*, and *SIX13* genes. Several *formae speciales* of *Fusarium oxysporum* including f. sp. *lycopersici*, f. sp. *canariensis*, f. sp. *lini*, f. sp. *cepae*, f. sp. *pisi*, f. sp. *freesia*, f. sp. *dianthi*, f. sp. *cubense*, and f. sp. *narcissi* have showed to contain *SIX10*, *SIX12*, and *SIX13* genes [12,17,22,54,55].

Even though, we found that the *SIX*-*Fum* genes profiles of *Fusarium* isolates were influenced by the geographical origin factor, the clustering of samples was not so clear. That low correlation is not very surprising when we know that nursery coffee seedlings circulate widely between localities and even between regions. We demonstrated that the *SIX*-*Fum* gene profiles are influenced by species (*F. oxysporum* vs. *F. solani*). The main differences between these two species were the absence of *SIX2* and *SIX6* in *F. solani* compared to *F. oxysporum*, whereas *SIX11* was present in at least one isolate of *F. solani* species but in *F. oxysporum* was not detected. The rest of the *SIX* genes except for *SIX7* and *SIX8* were overrepresented in the *F. solani* species in comparison with the percentages found in *F. oxysporum* isolates. It is well known that some *Fusarium* species different to the *F. oxysporum* species complex (FoSC) possess differential *SIX* gene profiles, this fact may be explained by the location of pathogenicity and toxin genes in mobile chromosomes which can be horizontally transferred, as it is the case of *SIX* and *Fum* genes [56,57]. These accessory chromosomes are involved in causing diseases by conferring advantages in specific environments, and virulence and pathogenic capacities on specific plant species [58].

We demonstrated an association between plant disease damages caused by the *Fusarium* isolates and their repertoire of *SIX* genes, as well as the presence of *Fum* 1 and *Fum* 13 genes. *Fum1* gene encodes the crucial iterative polyketide synthase (PKS) which starts the synthesis of the backbone of the mycotoxin fumonisin, whereas a reductase is encoded by *Fum13* gene [59]. Production of fumonisin has been reported for *F. oxysporum*, *F. graminearum*, *F. culmorum*, *F. equiseti*, *F. semitectum*, *F. fujikuroi*, *F. poae*, *F. subglutinans*, *F. verticillioides*, *F. proliferatum,* and *F. solani* [60,61,62,63]. In this study, we identified 20 *F. oxysporum* isolates and seven *F. solani* isolates as potential producers of fumonisin.

The isolate CBF-244 caused disease symptoms in coffee seedlings even though it contained no *SIX* and *Fum* genes, which means that its pathogenicity mechanisms do not involve the participation of these genes. Another interesting finding in this study was the presence of the *SIX7* gene in the isolates CBF-030, CBF-275, CBF-269, and CBF-277 that were of the most pathogenic. The presence of *SIX*7 has been demonstrated in *Fusarium oxysporum* isolates from Welsh onion seedlings, tomato, banana, and sesame [17,20,64,65]. It is possible that *SIX7* gene has an outstanding role in the pathogenicity showed in this study by *Fusarium* isolates associated with coffee corky-root disease.

In this study, we detected *F. solani* isolates containing at least one *SIX* gene. Recently, *SIX* genes have been identified in *Fusarium* species outside the FoSC including *F. proliferatum*, *F. hosta*e, *F. agapanthi* [66], *F. sacchari*, and *F. verticillioides* [55]. However, until now, *F*. *solani* had not been reported as a species containing effector *SIX* genes. The *F. solani* isolates CBF-265 and CBF-277 which contained *SIX* genes were pathogenic for coffee seedlings. This fact suggests that the presence of *SIX* genes in *F. solani* isolates could be related to their pathogenic capacities. Our results support the idea that the potential of colonization and development of pathogenicity of the *Fusarium* complex in coffee seedlings might be tightly related to the profile of *SIX* genes. Nevertheless, the existence of different mechanisms related to pathogenicity should not be ruled out for these isolates, since even though the *F. solani* isolate CBF-301 does not contain *SIX* genes but does contain *Fum* genes, it was pathogenic for coffee seedlings.

### 4.4. Host-Specificity of Fusarium oxysporum f. sp. lycopersici Results in Low Vascular Colonization without Causing Disease Symptoms in Coffee Seedlings

The *formae speciales* of *Fusarium oxysporum* are defined based on host specificity, and the subcategorization into races is dependent of their pathogenicity to a specific group of the host cultivars [67]. The ATCC 417, a *Fol* strain with high degree of virulence on tomato [68], was included in this study to determine if despite its host specificity, it was able to cause symptoms of disease in coffee seedlings. As we expected, the pathogenicity test revealed a low frequency of colonization by the ATCC 417 accompanied by its inability to cause symptoms of disease in coffee seedlings. Fourteen *SIX* genes have been identified in *Fol*, *SIX1*, *SIX3*, and *SIX5* directly contribute to virulence of *Fol* [38,54], whereas *SIX1*, *SIX3*, and *SIX4*, and interactions as *SIX3*–*SIX5* are recognized by the immune system and activate plant defense and resistance [69]. A specific *SIX* genes repertoire is associated with *Fusarium* isolates in the process of colonization and infection of a specific host [16,36]. In fact, host specificity of the phytopathogen *Fusarium oxysporum* f. sp. *lycopersici* is attributable to its lineage-specific mobile pathogenicity chromosomes in which are contained the secreted in xylem (*SIX*) genes [36]. 

## 5. Conclusions

Our investigation provides evidence: (i) about the association between the presence of *SIX* and *Fum* genes and the pathogenicity of *Fusarium* isolates linked to coffee corky-root disease. Future studies should be addressed to study the *SIX* genes transference intra- and inter-species. (ii) That some pathogenicity *SIX* genes and toxigenicity *Fum* genes seem to play a primordial role in the basic determinism of pathogenicity potential of *Fusarium* isolates on coffee, although in a complex and varied way for each of them depending on the isolate and independently of the species considered (*F*. *oxysporum* vs. *F*. *solani*). Transcriptomic studies quantifying the expression of these highlighted genes should allow to better understand this molecular basis of pathogenicity determinism in relation with the pathobiome environment. (iii) That *Fusarium solani* carries *SIX* and *Fum* genes, and because of its pathogenic abilities on coffee, appears as a potential pathogen contributing to the development of coffee corky-root disease. (iv) For the first time, the change in behavior and pathogenicity of *F*. *oxysporum* and *F*. *solani* isolates on coffee caused by water stress are demonstrated, which can be considered as mimicking the effect of root-knot-nematodes involved in corky-root disease. Efforts should focus in determining the function of the *Fusarium SIX* genes in coffee plant parasitism and its relationship with host specificity, as well as the mechanisms of other biotic and abiotic stresses activating the switch from hemi-biotrophic to necrotrophic phases in the pathogenic *Fusarium* species. Finally, crossed inoculations of the root-knot nematodes involved in the coffee corky-root disease, such as *M. paranaensis* or *M. arabicida*, with *F. oxysporum* and *F. solani* should be carried out to confirm the role of each of these causal agents in these pathogen complexes, and to better understand the interactions between them and with abiotic stresses in the etiology of this severe disease. (v) This work highlights the relevance of chlorophyll a fluorescence as an early and high-throughput phenotyping tool for plant pathogenicity studies on *Fusarium*. 

## Figures and Tables

**Table 1:** PCR screening targeting pathogenicity *SIX1*-*SIX14* genes, and two toxigenicity genes (*Fum1* and *Fum13*) for detection of putative fumonisin producing *Fusarium* isolates.

					*SIX* Genes															
*Fusarium* Species	Isolate Code	Accession	Municipality	Locality	1	2	3	4	5	6	7	8	9	10	11	12	13	14	*Fum*1	*Fum*13
*F. oxysporum*	CBF-297	CP053267.1	Atzalan	Chachalacas	+	-	-	-	-	-	-	-	-	-	-	-	-	-	-	-
*F. oxysporum* f. sp. *vasinfectum*	CBF-298	KT323848.1			-	-	-	-	-	+	+	-	-	-	-	-	-	-	-	-
*F. solani*	CBF-299	JF740846.1			-	-	-	-	-	-	-	+	-	-	-	-	-	-	-	-
*F. oxysporum*	CBF-032	KX822794.1		Napoala	+	-	-	-	-	+	-	-	-	-	-	-	-	-	-	-
*F. oxysporum*	CBF-033	KP964880.1			+	-	-	-	-	-	-	+	-	-	-	-	-	-	-	-
*F. oxysporum*	CBF-296	KP964859.1			+	-	-	+	+	+	+	-	-	-	-	-	-	-	+	+
*F. oxysporum*	CBF-027	KP964859.1	Cosautlán	La Lagunilla	-	-	-	-	-	+	+	+	-	-	-	-	-	-	+	+
*F. oxysporum*	CBF-038	KP964880.1			+	-	-	-	-	-	-	-	+	-	-	-	-	-	-	-
*F. oxysporum*	CBF-242	KP964900.1			-	-	-	-	-	-	-	-	+	-	-	-	-	-	-	-
*F. oxysporum*	CBF-243	KP964880.1			+	-	-	-	+	-	-	-	+	-	-	-	-	-	-	-
*F. oxysporum*	CBF-244 *	KP964880.1			-	-	-	-	-	-	-	-	-	-	-	-	-	-	-	-
*F. oxysporum*	CBF-308	CP053267.1			+	-	-	-	-	+	-	-	+	-	-	-	-	-	-	-
*F. oxysporum*	CBF-309	KP964859.1			-	+	-	+	-	+	+	+	-	-	-	-	-	-	-	-
*F. oxysporum*	CBF-245	KP964880.1	Ixhuatlán del café	Ocotitlán	+	-	-	+	-	-	-	+	-	-	-	-	-	-	-	-
*F. oxysporum*	CBF-246	KP964878.1			-	-	-	+	-	-	-	+	-	-	-	-	-	-	-	-
*F. oxysporum*	CBF-026	KP964880.1		Moctezuma	-	-	-	-	-	+	-	-	-	-	-	-	-	-	-	-
*F. oxysporum*	CBF-247	KP964880.1			+	-	-	+	-	-	-	-	-	-	-	-	-	-	-	-
*F. oxysporum*	CBF-248	KP964900.1			+	-	-	+	-	-	-	+	+	-	-	-	-	-	+	+
*F. oxysporum* f. sp. *vasinfectum*	CBF-249	KT323856.1			-	-	-	-	-	-	-	+	-	-	-	-	-	-	-	-
*F. oxysporum*	CBF-310	KP964880.1			-	-	-	-	-	-	-		-	-	-	-	-	-	-	-
*F. oxysporum* f. sp. *vasinfectum*	CBF-035	KT323856.1		Nevería	+	+	-	-	-	+	-	+	+	-	-	-	-	-	-	-
*F. oxysporum*	CBF-250	KP964900.1			+	-	-	+	-	-	-	-	-	-	-	-	-	-	-	-
*F. oxysporum*	CBF-251	KP964880.1			-	-	-	+	-	+	-	+	-	-	-	-	-	-	-	-
*F. oxysporum*	CBF-252	KP964880.1			-	-	-	-	-	-	-	+	-	-	-	-	-	-	-	-
*F. oxysporum*	CBF-253	KP964900.1			-	-	-	-	-	-	-	-	-	-	-	-	-	-	-	-
*F. oxysporum* f. sp. *vasinfectum*	CBF-254 *	KT323838.1			-	-	-	+	-	-	-	+	+	-	-	-	-	-	-	-
*F. oxysporum*	CBF-305	KP964880.1			-	-	-	-	-	+	+	-	-	-	-	-	-	-	-	-
*F. oxysporum* f. sp. *vasinfectum*	CBF-306	KT323856.1			+	-	-	-	-	+	-	-	+	-	-	-	-	-	-	-
*F. oxysporum*	CBF-307	CP053267.1			-	-	-	-	-	+	-	-	+	-	-	-	-	-	-	-
*F. oxysporum*	CBF-255	CP053267.1	Emiliano Zapata	Pacho Nuevo	+	-	-	-	+	-	+	+	-	-	-	-	-	+	+	+
*F. oxysporum*	CBF-256	KP964880.1			+	-	-	-	+	-	-	+	-	-	-	-	-	-	-	-
*F. oxysporum* f. sp. *cepae*	CBF-257	KP964904.1			-	-	-	+	-	-	+	+	-	-	-	-	-	-	-	-
*F. oxysporum* f. sp. *vasinfectum*	CBF-258	KT323846.1			-	-	+	-	-	-	+	+	+	-	-	-	-	+	+	+
*F. oxysporum*	CBF-028	KP964880.1	Jilotepec	Paso San Juan	-	-	-	-	-	-	+	-	-	-	-	-	-	-	+	+
*F. oxysporum*	CBF-030	CP053267.1			-	+	-	-	-	+	+	-	-	-	-	-	-	-	+	+
*F. oxysporum*	CBF-031	KP964880.1			-	-	-	-	-	-	+	-	-	-	-	-	-	-	+	+
*F. oxysporum* f. sp. *dianthi*	CBF-259	LT841231.1			-	-	-	+	-	-	+	+	-	-	-	-	-	+	-	-
*F. oxysporum* f. sp. *vasinfectum*	CBF-260	KT323848.1			-	-	+	-	+	-	-	-	-	-	-	-	-	-	+	+
*F. oxysporum* f. sp. *dianthi*	CBF-261	LT841231.1			+	-	-	+	-	-	+	-	+	-	-	-	-	-	+	+
*F. oxysporum*	CBF-262	KP964878.1			+	-	+	+	-	-	+	-	-	-	-	-	-	-	+	+
*F. solani*	CBF-263 *	JF740784.1			-	-	+	+	-	-	-	+	+	-	+	-	-	+	+	+
*F. oxysporum*	CBF-036	KP964880.1	Sochiapa	Sochiapa	+	+	+	-	-	-	-	-	-	-	-	-	-	-	-	-
*F. oxysporum*	CBF-037	KP964859.1			-	-	-	-	+	-	-	+	-	-	-	-	-	-	+	+
*F. solani*	CBF-264	JF740846.1			+	-	-	-	+	-	-	-	-	-	-	-	-	-	+	+
*F. solani*	CBF-265	JF740846.1			+	-	-	+	+	-	-	+	+	-	-	-	-	+	-	-
*F. oxysporum*	CBF-266	KP964880.1			-	-	-	-	-	-	+	-	-	-	-	-	-	-	-	-
*F. oxysporum* f. sp. *phaseoli*	CBF-267	KP964890.1			+	-	-	-	-	+	-	-	-	-	-	-	-	+	-	-
*F. oxysporum*	CBF-268	KP964880.1			-	-	-	+	-	-	+	-	-	-	-	-	-	+	+	+
*F. oxysporum*	CBF-269 *	KP964859.1			-	-	-	+	-	-	+	+	-	-	-	-	-	-	+	+
*F. oxysporum* f. sp. *dianthi*	CBF-292	LT841231.1			-	-	-	-	-	+	+	-	-	-	-	-	-	-	-	-
*F. oxysporum*	CBF-293	KP964859.1			-	-	-	+	+	+	+	+	-	-	-	-	-	-	+	+
*F. solani*	CBF-294	JF740846.1			+	-	-	-	-	-	-	+	-	-	-	-	-	-	-	-
*F. oxysporum* f. sp. *vasinfectum*	CBF-295	KT323856.1			-	-	-	-	-	-	-	-	+	-	-	-	-	-	-	-
*F. oxysporum* f. sp. *vasinfectum*	CBF-270	KT323869.1	Totutla	Totutla	-	-	-	-	+	-	-	+	+	-	-	-	-	-	-	-
*F. oxysporum* f. sp. *vasinfectum*	CBF-271	KT323869.1			-	-	+	-	+	+	+	+	+	-	-	-	-	+	+	+
*F. oxysporum*	CBF-272	KP964859.1			-	-	+	-	-	-	-	+	-	-	-	-	-	-	-	-
*F. solani*	CBF-273	JF740784.1	Coatepec	Tuzamapan	+	-	+	-	+	-	+	+	+	-	-	-	-	+	+	+
*F. oxysporum*	CBF-274	KP964880.1			-	-	+	-	+	-	-	-	+	-	-	-	-	-	-	-
*F. oxysporum*	CBF-275	KP964880.1			+	-	+	-	+	+	+	-	+	-	-	-	-	+	+	+
*F. solani*	CBF-276	JF740846.1			+	-	-	-	+	-	-	-	+	-	-	-	-	+	-	-
*F. solani*	CBF-277	JF740846.1			+	-	+	+	+	-	+	+	-	-	-	-	-	+	+	+
*F. oxysporum* f. sp. *vasinfectum*	CBF-278	KT323848.1			+	-	+	-	+	-	-	+	-	-	-	-	-	-	-	-
*F. solani*	CBF-279	JF740727.1			+	-	+	+	+	-	-	-	-	-	-	-	-	-	+	+
*F. oxysporum*	CBF-280	KP964878.1			+	-	-	-	+	-	-	+	-	-	-	-	-	-	-	-
*F. oxysporum*	CBF-281	KP964880.1		Zimpizahua	+	-	-	-	-	-	+	-	-	-	-	-	-	-	+	+
*F. solani*	CBF-282	JF740846.1			+	-	+	+	+	-	-	-	-	-	-	-	-	-	+	+
*F. oxysporum*	CBF-283	KP964878.1			-	-	-	+	-	+	+	-	-	-	-	-	-	-	-	-
*F. oxysporum*	CBF-034	KP964859.1	Yecuatla	La Victoria	-	-	-	-	-	+	-	+	-	-	-	-	-	-	+	+
*F. solani*	CBF-301	JF740786.1			-	-	-	-	-	-	-	-	-	-	-	-	-	-	+	+
*F. oxysporum* f. sp. *phaseoli*	CBF-303	KP964890.1			-	-	-	-	-	+	+	-	-	-	-	-	-	-	+	+
*F. oxysporum* f. sp. *lycopersici*	ATCC 417				+	+	+	+	+	+	+	+	-	-	+	-	-	-	-	+

+, Presence of *SIX* gene; -, absence of *SIX* gene; *, *Fusarium* isolates from healthy coffee seedlings.

**Table 2:** Correlations between variables and dimensions, eigenvalues, and percentage of variance for the first four dimensions resulting from the PCA analysis on photosynthetic variables.

	PC1	PC2	PC3	PC4
PI_total_	−0.90	0.21	0.31	−0.15
PI_abs_	−0.94	0.26	−0.01	−0.06
Fv/Fm	−0.93	−0.13	−0.31	0.01
ABS/RC	0.98	0.11	0.05	0.12
Di_0_/RC	0.94	0.26	0.06	0.18
Tr_0_/RC	−0.31	−0.93	0.17	0.10
Et_0_/RC	−0.94	0.09	−0.04	0.29
phi(Eo)	−1.00	−0.01	−0.01	−0.02
ψ_0_/Vj	−0.91	0.28	0.13	0.20
Eigenvalues	7.20	1.16	0.25	0.21
Variance (%)	79.95	12.89	2.73	2.35

## Supplementary Materials

The following figures are available online at https://www.mdpi.com/article/10.3390/jof7040253/s1, Figure S1: Three dimensional non-metric multidimensional scaling (3D-NMDS) ordination of the *Fusarium* isolates associated to coffee corky-root disease, Figure S2: Spearman correlation plot between disease damage and physiological parameters of coffee seedlings.
