# Supplementary material for: Molecular and Environmental Triggering Factors of Pathogenicity of Fusarium oxysporum and F. solani Isolates Involved in the Coffee Corky-Root Disease
Source: J Fungi (Basel). 2021 Mar 27;7(4):253. doi: 10.3390/jof7040253 (PMC8067267; doi:10.3390/jof7040253)
Supplement: Supplementary file 1 [file jof-07-00253-s001.pdf]

Supplementary Materials:

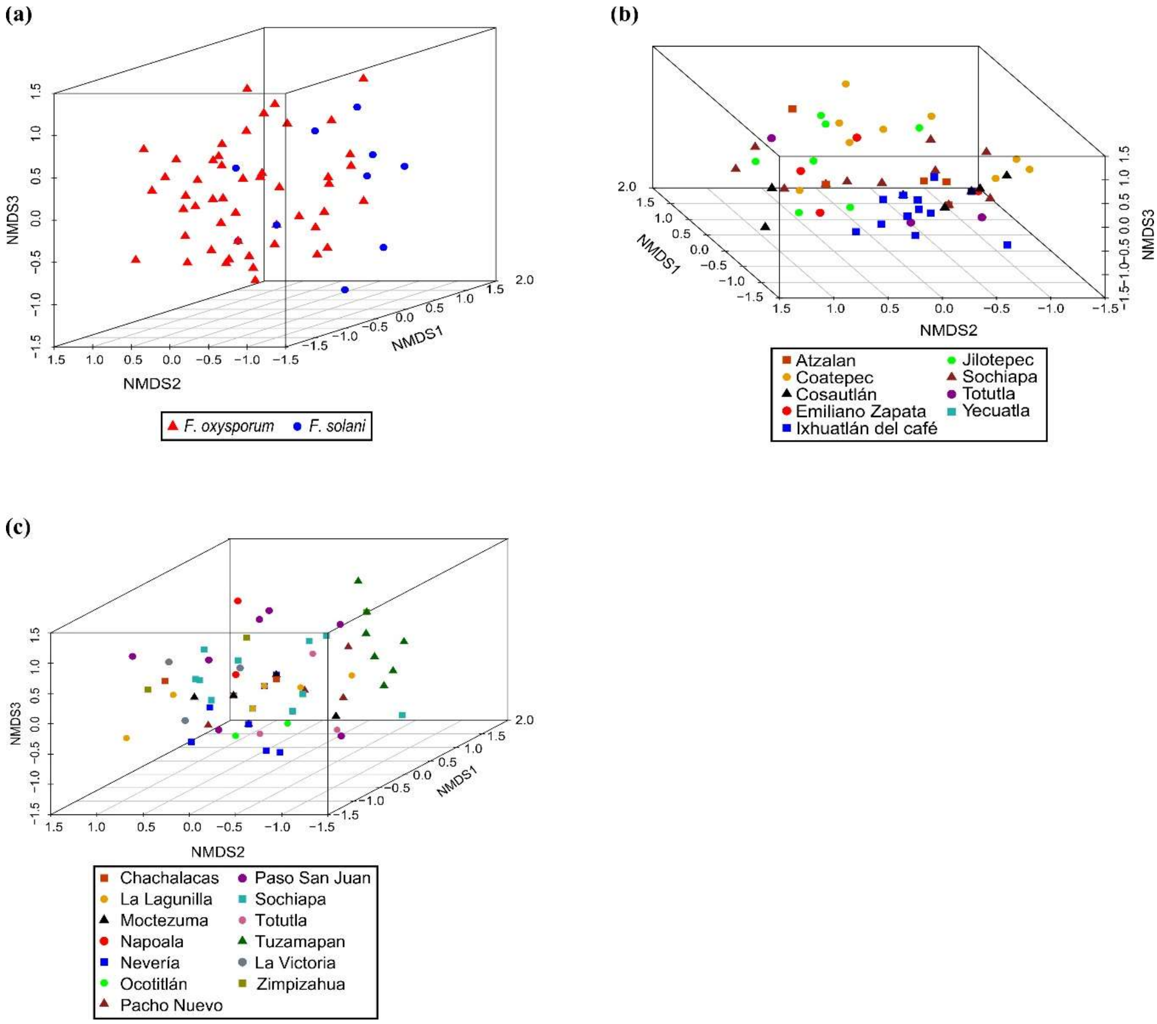

Figure S1. Three dimensional non-metric multidimensional scaling (3D-NMDS) ordination of the *Fusarium* isolates associated to coffee corky-root disease. 3D stress value = 0.10. 3D-NMDS plot annotated by: (**a**) specie; (**b**) municipality; and (**c**) locality.

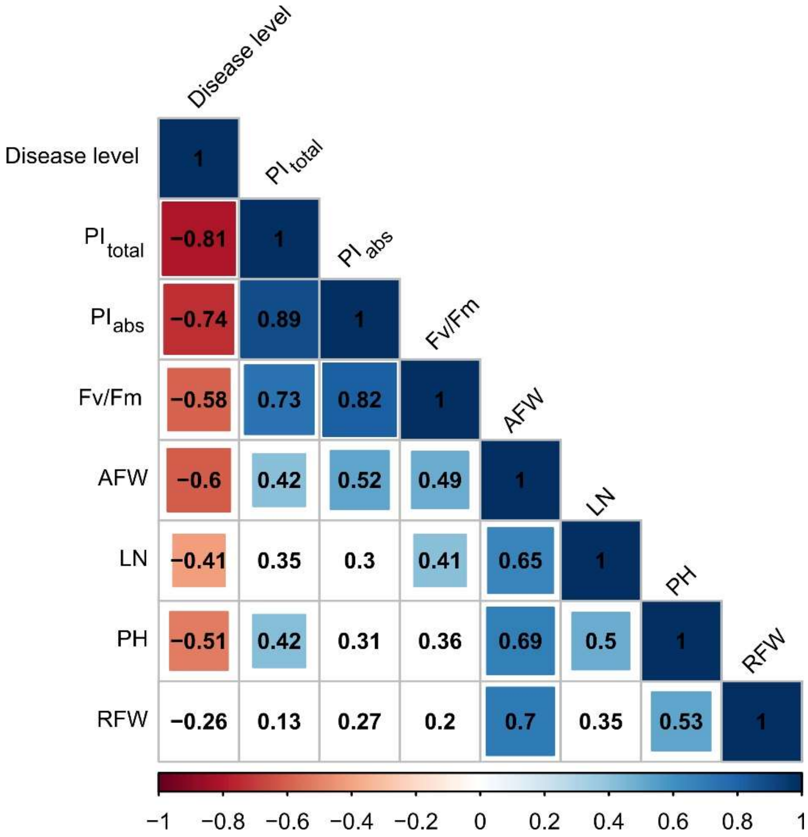

Figure S2. Spearman correlation plot between disease damage and physiological parameters of coffee seedlings. The Spearman correlation coefficients are depicted. Statistical significance: $P < 0.01$. White squares represent no significant correlations.
